# Sleep disorders in children with moderate to severe persistent allergic rhinitis^☆^

**DOI:** 10.1016/j.bjorl.2017.01.008

**Published:** 2017-02-27

**Authors:** Jessica Loekmanwidjaja, Ana Cláudia F. Carneiro, Maria Lúcia T. Nishinaka, Daniela A. Munhoes, Gabriela Benezoli, Gustavo F. Wandalsen, Dirceu Solé

**Affiliations:** Universidade Federal de São Paulo (UNIFESP), Escola Paulista de Medicina (EPM), Departamento de Pediatria, São Paulo, SP, Brazil

**Keywords:** Sleep disorders, Allergic rhinitis, Children, Written questionnaire, Distúrbios de sono, Rinite alérgica, Crianças, Questionário escrito

## Abstract

**Introduction:**

Allergic rhinitis is associated with several complications, including sleep disorders. The Children's Sleep Habits Questionnaire has been recently translated and validated in Portuguese for the evaluation of sleep disorders in children.

**Objective:**

To assess sleep disorders in children with moderate to severe persistent allergic rhinitis and to correlate the findings with disease severity markers.

**Methods:**

We evaluated 167 children (4–10 years), 112 with allergic rhinitis and 55 controls. Parents/guardians of the children answered the Children's Sleep Habits Questionnaire, consisting of 33 questions divided into eight subscales, which refers to the previous week. Patients with rhinitis were also evaluated regarding the score of nasal and extra-nasal symptoms related to the previous week and the peak nasal inspiratory flow.

**Results:**

There were no significant differences between groups of different age. All patients with rhinitis were being treated with nasal topical corticosteroids. The total Children's Sleep Habits Questionnaire score was significantly higher among children with rhinitis than in controls (median 48 vs. 43, *p* < 0.001). Significantly higher values were also observed for the parasomnia (9 vs. 8), respiratory disorders (4 vs. 3) and daytime sleepiness (14 vs. 12) subscales. Among the patients with rhinitis, no significant correlation was observed between the total Children's Sleep Habits Questionnaire score and disease activity variables, but moderate correlations were observed for the respiratory distress subscale vs. nasal symptom score (*r* = 0.32) and vs. extra-nasal symptom score (*r* = 0.32).

**Conclusion:**

Children with moderate to severe persistent allergic rhinitis, even when submitted to regular treatment, have a higher frequency of sleep disorders than controls, particularly concerning nocturnal breathing disorders, daytime sleepiness, and parasomnias. The intensity of sleep disorders found in some subscales was correlated with objective markers of allergic rhinitis severity.

## Resumo

**Introdução:**

A rinite alérgica está associada a diversas complicações, como por exemplo os distúrbios do sono. O *Children's Sleep Habits Questionnaire* é um questionário para avaliação dos distúrbios do sono em crianças, recentemente traduzido e validado para o português.

**Objetivos:**

Avaliar distúrbios do sono em crianças com rinite alérgica persistente moderada/grave e correlacionar os achados com marcadores de gravidade da doença.

**Método:**

Foram avaliadas 167 crianças (4–10 anos), 112 com rinite alérgica e 55 controles. Todos os responsáveis pelas crianças responderam o questionário composto por 33 questões dividas em oito subescalas e referentes à última semana. Os pacientes com rinite foram avaliados também pelo escore de sintomas nasais e extra-nasais referentes à última semana e pelo pico de fluxo inspiratório nasal.

**Resultados:**

Não houve diferenças significantes entre os grupos com relação à idade. Todos os pacientes com rinite vinham sendo tratados com corticosteroide tópico nasal. O escore total do questionário foi significantemente maior entre os com rinite que os controles (mediana 48 vs. 43; *p* < 0,001). Valores significantemente maiores também foram observados para as subescalas de parassonias (9 vs. 8), distúrbios respiratórios (4 vs. 3) e sonolência diurna (14 vs. 12). Entre os pacientes com rinite não foi observada correlação significante entre o escore total do questionário e as variáveis de atividade da doença, porém correlações moderadas foram observadas para a subescala de distúrbios respiratórios vs. escore de sintomas nasais (r = 0,32) e vs. escore de sintomas extra-nasais (r = 0,32).

**Conclusões:**

Crianças com rinite alérgica persistente moderada-grave, mesmo em tratamento regular, apresentam maior frequência de distúrbios do sono do que controles, particularmente em relação aos distúrbios respiratórios noturnos, sonolência diurna e parassonias. A intensidade das alterações do sono encontradas em algumas subescalas se correlacionou com marcadores objetivos de gravidade da rinite alérgica.

## Introduction

Data obtained by the International Study of Asthma and Allergies in Childhood (ISAAC) indicate that the prevalence of rhinitis symptoms in Brazilian schoolchildren and adolescents is 25.7% and 29.6%, respectively, and that of allergic rhinoconjunctivitis is 12.6% for children and 14.6% for adolescents.^1^

Although allergic rhinitis is often viewed as a disease of lesser severity than asthma, it is capable of dramatically altering the patients’ quality of life, as well as their performance, learning and productivity.^2^, ^3^, ^4^, ^5^, ^6^, ^7^ In addition to symptoms themselves, the treatment regimen may be blamed for the discomfort reported by patients with allergic rhinitis, especially those with more severe forms.^8^ Sleep disorders, difficulty concentrating, decreased performance (school/work) and daytime sleepiness have often been reported by patients with allergic rhinitis, especially in the persistent forms.^2^, ^3^, ^4^, ^5^, ^6^, ^7^, ^8^

The evaluation of the interference of allergic rhinitis on sleep has been the object of study by researchers; however, the use of objective methods, such as polysomnography, is very limited in population studies due to technical and practical difficulties. Thus, the development of questionnaires and assessment scales to be utilized in pediatric populations has been developed for use in larger studies.^9^, ^10^, ^11^, ^12^, ^13^, ^14^, ^15^, ^16^ Among these, the use of the Children's Sleep Habits Questionnaire (CSHQ) is emphasized,^17^ the purpose of which is to assess sleep behavior in school-age children including the most common symptoms of sleep disorders in children, according to the International Classification of Sleep Disorders.^18^ Developed in the English language, the CSHQ was recently translated and validated into Portuguese and a mean global score of 47 points was observed among healthy children.^19^

To date, only a single study has assessed the presence of sleep disorders in children with respiratory allergies using the CSHQ questionnaire.^4^ In this multicenter study, carried out in several Latin American centers, it was demonstrated that children with asthma and/or allergic rhinitis had a higher prevalence of sleep disorders when compared to controls.^4^

The aim of this study was to evaluate the presence of sleep disorders in children with moderate to severe persistent allergic rhinitis and to correlate these findings with disease severity markers.

## Methods

Patients (aged 4–10 years) enrolled were regularly followed for at least one year in an outpatient clinic due to Persistent Moderate-to-Severe Allergic Rhinitis (PMSAR). Healthy, non-allergic controls of the same age group also were enrolled in the study. All children underwent a complete physical examination at admission to rule out possible chronic diseases capable of interfering with sleep quality, in addition to medication consumption. Children (patients and controls) with upper airway mechanical obstruction, neurological diseases, psychiatric disorders or those taking anticonvulsants, as well as those with uncontrolled asthma, were excluded,^20^ including children receiving first-generation antihistamines.

The childrens’ parents/guardians answered the CSHQ, which was translated and validated into the Portuguese language.^19^ The CHSQ consists of 33 questions divided into subscales as follows: resistance to sleep (goes to bed at the same time, falls asleep only in his/her own bed, falls asleep in someone else's bed, needs a parent in the bedroom, is reluctant to go to sleep (score: 6–18 points); start of sleep (score: 1–3 points), sleep duration (sleeps little, sleeps the necessary hours, sleeps the same number of hours every day – score: 3–9 points); sleep anxiety (needs a parent in the bedroom, fear of sleeping in the dark, fear of sleeping alone, problems sleeping outside the home – score: 4–12 points); nocturnal awakenings (switches to someone else's bed in the middle of the night; wakes up once a night, wakes up more than once – score: 3–9 points); parasomnias (wets the bed at night, talks during sleep, is restless and tosses or thrashes during sleep; sleepwalks; teeth grinding during sleep; wakes up screaming, sweating; wakes up scared after a nightmare – score: 7–21 points); respiratory sleep disorders (breathes loudly; seems to stop breathing during sleep, snoring – score: 3–9 points) and daytime sleepiness (wakes up alone in the morning, wakes up in a bad mood, is awakened by others, has difficulty getting out of bed, is slow to be fully awake, seems tired when: watching TV, riding a car – score: 8–24 points). The score is obtained by adding the points referring to the questions and the total score (range: 35–105 points) by adding the scores of the eight items.^17^

Patients with PMSAR were also evaluated for clinical nasal symptom score (NSS – nasal obstruction, nasal pruritus, sneezing and rhinorrhea) and extra-nasal symptom score (ENSS, ocular hyperemia, ocular pruritus, tearing and pharyngeal pruritus) in relation to the previous week. A score was attributed to each symptom, varying from 0 to 3 (0 = absent, 1 = mild, 2 = moderate and 3 = intense).^21^ Thus, total NSS and total ENSS ranged from 0 to 12 points. Patients with NSS ≤ 3, when not related to a single symptom, were considered to have controlled rhinitis.

In addition to the scores, patients with PMSAR were evaluated for nasal cavity function by measuring the Peak Nasal Inspiratory Flow (PNIF) using a specific device (Peak Nasal Inspiratory Flow Meter^®^, HS Clement Clarke, UK). During the procedure, patients, after maximal lung expiration, with the peak flow meter coupled to the face, were instructed to perform maximal inspiration through the nose, while keeping the lips fully closed, up to the total lung capacity. Three measurements were made and the best was recorded, since there was no difference greater than 10% among them.^22^ The maximum nasal inspiratory flow was recorded by the device cursor in liters per minute.

The study was approved by the institution's Research Ethics Committee (824,192/2014). The children's parents and/or guardians, as well as the children, agreed to participate in the study by signing the informed consent form, as well as the assent form for the children.

### Statistical analysis

Parametric and nonparametric tests were used according to the nature of the assessed variables. The comparison between the ages of patients and controls was carried out by Student's *t*-test. The comparative analysis of total scores and subscales between patients and controls and between those with controlled and uncontrolled rhinitis was performed using the Mann–Whitney test. The study of the association between the global scores and the CHSQ subscales and the NSS and the PNIF was performed using Spearman's correlation coefficient. In all tests, the level of rejection for the null hypothesis was set at 5%.

The sample calculation was performed considering the mean of the CSHQ total score of 47 points observed in the validation of the Portuguese questionnaire,^19^ a minimum difference of 4 points in relation to the controls, standard deviation of 8 points, 80% power and *p* = 0.05. This would require 51 children per group. To study the correlation of the questionnaire with the allergic rhinitis severity variables, we considered a minimum correlation coefficient of 0.25, power of 80% and *p* = 0.05; which required at least 98 children in the group with allergic rhinitis.

## Results

A total of 112 children with AR and 55 control children completed the study. The two groups were similar in age (Table 1). Among the patients, 48 (43%) were females; 88 (79%) had asthma and 8 (7%) had allergic conjunctivitis associated with PMSAR. All patients were being treated with nasal topical corticosteroids and some of them, 46 (41%) received second-generation oral H1 antihistamine in the week preceding the evaluation. PNIF measurement was appropriately performed in 94% (94/112) of the patients.

**Table 1:** Comparative analysis of children with moderate to severe persistent allergic rhinitis and non-allergic controls according to the total score and subscale score (median and interquartile range) of the Children's Sleep Habits Questionnaire (CSHQ).

	Control	Allergic rhinitis	*p*
*N*	55	112	
Age (years)^b^	7.3 ± 1.5	8.0 ± 1.8	0.2
Total CSHQ score^c^	43 (38–49)	48 (44–54)^a^	<0.0001
Subscales			
Resistance to sleep^c^	7 (6–10)	7 (6–10)	0.7
Start of sleep^c^	1 (1–2)	1 (1–2)	0.4
Sleep duration^c^	3 (3–3)	3 (3–5)	0.06
Sleep anxiety^c^	5 (4–7)	6 (4–8)	0.3
Nocturnal awakenings^c^	3 (3–4)	4 (3–4)	0.08
Parasomnias^c^	8 (7–10)	9 (8–10)^a^	0.002
Respiratory disorders^c^	3 (3–4)	4 (3–5)^a^	0.003
Daytime sleepiness^c^	12 (9–14)	14 (10–17)^a^	0.003

a Median and interquartile interval–significantly higher.

b Student's *t*-test.

c Mann–Whitney.

The comparative analysis between the two groups in relation to CSHQ total score showed significantly higher values of total scores and scores of the parasomnia, respiratory disorders, and daytime sleepiness subscales in PMSAR patients (Table 1).

The presence of asthma among the patients did not induce significant changes in the total score and those of the subscales when compared to those with PMSAR alone (total rhinitis with asthma score vs. rhinitis without asthma: Median [Me] = 49; interquartile interval [IQI] = 43–54) vs. Me = 47 [IQI = 44–53]; *p* = 0.8).

**Figure 1:**
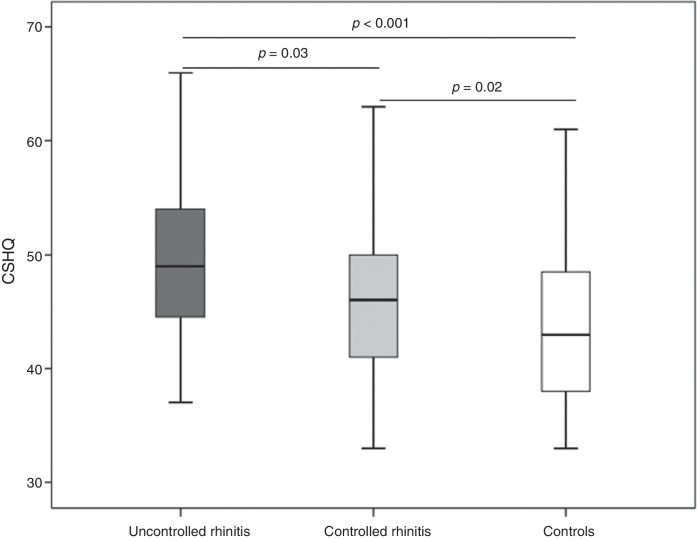
Total score of Children's Sleep Habits Questionnaire (CSHQ) in children with controlled allergic rhinitis (controlled rhinitis: nasal symptom score ≤3) (light gray), uncontrolled (uncontrolled rhinitis: nasal symptom score ≥4) (dark gray) and controls (white).

Using the NSS >3 criterion to classify PMSAR as uncontrolled, we found that 53 (47.3%) patients had uncontrolled PMSAR. The total CSHQ score was significantly higher among those with uncontrolled rhinitis (Me = 49; IQI = 44–54) when compared to those with controlled rhinitis (Me = 46; IQI = 41–50) (Fig. 1). These two subgroups of children with allergic rhinitis showed significantly higher scores than those in the control group (Fig. 1). Similarly, the use of oral antihistamines was not associated with changes in the questionnaire responses (total score use versus non-use: Me = 46 [IQI = 42–52] vs. Me = 49 [IQI = 44– 53], *p* = 0.2).

Considering the mean of the CSHQ total score observed in its Portuguese validation (47 points) as a criterion for sleep disorder diagnosis, we found that among patients with PMSAR, 57 (51%) could be considered as having sleep disorders. Regarding the control of rhinitis, 58% of those with uncontrolled rhinitis showed alterations in the total CSHQ score, in comparison with 44% of those with controlled rhinitis.

**Figure 2:**
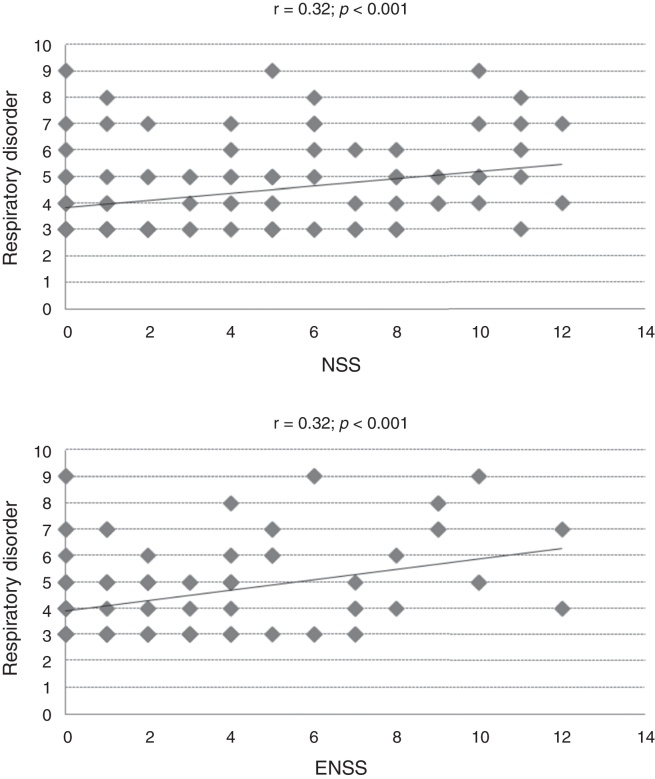
Dispersion of values in the Children's Sleep Habits Questionnaire (CSHQ) specific subscale and allergic rhinitis control markers (NSS, nasal symptom score, ENSS, extra-nasal symptom score, *r* = Spearman's correlation coefficient).

The study of the association between the total CSHQ score and its subscales with NSS, ENSS and PNIF values documented the partial significance of these comparisons (Table 2). Moderate correlation coefficients (*r* > 0.30) were observed only between the respiratory disorder subscale and nasal (*r* = 0.32) and extra-nasal (*r* = 0.32) symptom scores (Table 2, Fig. 2).

**Table 2:** Spearman's correlation coefficients (significant values: *p* < 0.05) between the total score and the subscale scores of the Children's Sleep Habits Questionnaire (CSHQ) and allergic rhinitis control markers.^a^

	NSS	ENSS	PNIF
	*r*		
Total CSHQ score	0.15	0.16	−0.22
Nocturnal awakenings	–	0.16	–
Parasomnias	–	0.25	–
Respiratory disorders	0.32	0.32	–
Daytime sleepiness	0.17	–	−0.27

a NSS, nasal symptom score; ENSS, extra-nasal symptom score; PNIF, peak nasal inspiratory flow.

## Discussion

It is believed that allergic rhinitis can affect sleep through different mechanisms. Nasal congestion secondary to the nasal mucosa inflammatory process induces increased airway resistance and may result in oral breathing, sleep disruption and fatigue.^23^ Additionally, inflammatory mediators of the allergic process, such as histamine and certain cytokines, can act directly on the central nervous system by altering sleep rhythm.^23^ It has been recently observed, in children with sleep disorders, that the presence of allergic rhinitis (without obstructive sleep apnea) decreases REM (Rapid Eye Movement) sleep time.^24^

Although considered as one of the main consequences of allergic rhinitis, sleep alterations or disorders have been minimally evaluated by tools validated in children.^25^

In our study, we demonstrated that children with allergic rhinitis showed higher scores on CSHQ when compared to healthy children, indicating a greater chance of sleep disorders observed in them. However, if we consider the isolated use of CSHQ, which score would be able to identify the presence of sleep disorders? According to the creators of the CSHQ, this score would be 41.^17^ Using this value as a cutoff, we found that 83% of the children with PMSAR would be classified as having sleep disorders and among the controls, that percentage would reach 60%. On the other hand, if we used the median of the total CSHQ score observed in our control group (Me = 43) as the cutoff, we would have 76% of sleep alterations among those with PMSAR and 44% in controls. However, this cutoff was 47 in the large validation study for the Portuguese language.^19^ By assuming this cutoff, we would have sleep disorders in 51% of those with PMSAR and in 24% of the controls. In view of these results, whatever the criteria used, it is clear that patients with PMSAR have a higher frequency of sleep disorders.

A recent systematic review evaluated the association between allergic rhinitis and sleep disorders in children.^26^ Of the 18 selected studies published in the last 25 years, 12 found a higher prevalence of sleep disorders in children with allergic rhinitis, with a prevalence of habitual snoring.^26^ The broad differences in the methods used in each study, involving different age groups and different diagnostic methods, did not allow the accomplishment of a meta-analysis and compilation of the review data,^26^ as well as making it difficult to compare it with our study.

Several studies have shown that nasal congestion is an independent risk factor for respiratory disorders during sleep. Sleep is more affected when nasal congestion is severe, leading to parasomnias, snoring, breathing disorders and, consequently, daytime sleepiness.^26^ Our study demonstrated that parasomnias, respiratory disorders, and daytime sleepiness have higher medians in patients with allergic rhinitis than in control patients. These sleep disorders observed in children with allergic rhinitis probably contribute to the previously described complications of allergic rhinitis, such as decrease in quality of life and inadequate school performance.^2^, ^3^, ^4^, ^5^, ^6^, ^7^

The present study showed a significant association between sleep disorders and several markers of allergic rhinitis severity or lack of control, a finding documented to date by a limited number of studies.^25^ Significantly higher CSHQ scores were found in those with uncontrolled allergic rhinitis (Fig. 1) and there was a significant correlation between several subscales of the questionnaire and symptom scores and peak nasal flow (Table 2). These correlations, however, were weak in most cases, with moderate correlations found only in the respiratory disorder subscale.

In our study, an objective tool for evaluating nasal function was used: the peak nasal inspiratory flow. We were able to document a significant and negative correlation of this variable with the total score of the questionnaire and the daytime sleepiness subscale.

The lack of regular and effective treatment of allergic rhinitis has been indicated as one of the probable causes of the association between the disease and sleep disorders.^26^ Clinical trials have observed that the introduction of the intranasal corticosteroid in these patients positively contributes to the quality of sleep and the reduction of these disorders.^27^, ^28^ Our study included only patients receiving intranasal corticosteroid treatment and we demonstrated that the association of rhinitis with sleep disorders is also observed in these children. It is noteworthy the high rate of treated and yet uncontrolled patients (47%) exists. This finding may be due, in part, to the strict criteria defined for the control of allergic rhinitis (symptom score of up 3 points on a 12-point scale). On the other hand, studies in patients with allergic rhinitis have documented the persistence of symptoms in many of them, even with regular use of medication.^3^, ^29^ Finally, no objective medication use control was carried out, since this was not the purpose of the study and, therefore, it is not possible to guarantee real and regular medication use.

Although widely used, the CSHQ still lacks validation against classical tools for the diagnosis of sleep disorders. To the best of our knowledge, only one study compared CSHQ subscales with objective findings demonstrated in children's polysomnography.^30^ In this study, no significant correlation was found between the polysomnography results and four subscales of the CSHQ. The authors observed that these questionnaire subscales show low sensitivity and high specificity in the diagnosis of sleep disorders.^30^ However, it is important to observe that sleep disturbances encompass a wide range of disorders, diseases and symptoms. The questionnaire used in the present study (CSHQ) is a screening tool, a limitation of which is not being able to conclusively establish the presence or absence of sleep disorders and their characteristics.

The presence of other allergic diseases, such as asthma, or treatments with certain medications could represent a possible study bias. To minimize this fact, we chose to include only children with controlled asthma and exclude those receiving anticonvulsants and classical antihistamines. We observed that the presence of controlled asthma and the use of second-generation antihistamines were not significantly associated with the questionnaire responses.

## Conclusion

Children with PMSAR, even when submitted to regular treatment, have a higher frequency of sleep disorders than controls, particularly in relation to nocturnal respiratory disorders and daytime sleepiness. The intensity of sleep disturbances found in these subscales correlated with objective markers of allergic rhinitis severity.

## Conflicts of interest

The authors declare no conflicts of interest.
